# Editorial: Multidimensional interplay of early-life events, neuroactive steroids and sex in the development of psychopathology and psychiatric disorders, volume II

**DOI:** 10.3389/fnbeh.2024.1360064

**Published:** 2024-01-15

**Authors:** Liana Fattore, Roberto Frau, Fabrizio Sanna

**Affiliations:** ^1^Institute of Neuroscience-Cagliari, Consiglio Nazionale delle Ricerche (CNR), Pisa, Italy; ^2^Department of Biomedical Sciences, University of Cagliari, Cagliari, Italy; ^3^Guy Everett Laboratory, Department of Biomedical Sciences, University of Cagliari, Cagliari, Italy

**Keywords:** stress, sex differences, psychiatric disorders, neurosteroids, early-life adversities

The first volume of the Research Topic titled ‘*Multidimensional interaction of early life events, neuroactive steroids, and sex in the development of psychopathology and psychiatric disorders'* has garnered an excellent response, resulting in the publication of numerous original articles and reviews. By the end of 2023, more than 23,000 readers have engaged with the content, and 5,124 downloads have been recorded.

We believe that the considerable interest in this topic stems from a growing awareness that the origins of psychiatric disorders require a comprehensive examination of various factors, including sex/gender, substance use, and early-life adverse events. As emphasized in the first volume, beyond the polygenic risk of mental diseases, both preclinical and clinical studies have identified numerous environmental exposures contributing to the etiology of mental illnesses. More importantly, these early-life exposures shape the pathophysiological trajectory of major psychiatric disorders in a sex-dependent manner, underscoring the prominent role of sex hormones in the interplay between early-life events and mental health.

Therefore, to achieve a profound understanding of the biological basis of psychiatric diseases and to pave the way for the development of effective prevention and intervention strategies, it is imperative to consider the contributions of sex and environmental factors in both clinical investigations and experimental models.

The first three articles of our Research Topic focus on the role of hormones as an early intervening factor in fostering developmental pathological trajectories. The article by Johnston and Wanat reviews the link between early social isolation and development of social anxiety disorder (SAD) in humans and rodent preclinical models and the potential involvement of the neuropeptide oxytocin. The key role of oxytocin in social interactions is unanimously recognized as well as the presence of impairments in its function in several pathological conditions affecting social behaviors. However, the precise mechanisms involved in these alterations and the emerging possibility of using oxytocin as a therapeutic agent are still poorly understood, especially when considering the problem from a developmental perspective. Also highlighted is the importance of taking into account the role(s) of oestrogens and androgens in light of their strict interactions with oxytocin, possibly at the basis of the sex/gender-related differences observed in early social isolation-induced SAD.

The review by Mbiydzenyuy et al. aims at identifying the (neuro)biological alterations that characterize prenatal maternal stress and their relationship with the development of aggressive behavior in the offspring. Three main mechanisms are hypothesized: (i) dysregulations related to maternal-fetal HPA axis functioning, (ii) vascular alterations related to intrauterine environment and iii) epigenetic modifications through which maternal stress could inter- and transgenerationally pass to the offspring and affect behavior. In particular, the relationship between epigenetic mechanisms and the neuroendocrine and brain changes that characterize aggressive behavior in the offspring are analyzed. Epigenetic changes induced by negative events in the mother may provide a molecular basis for the aggressive traits observed in the offspring, highlighting the transcriptional potential that prenatal stress can have and how these modifications can long-lastingly affect aggression and, more generally, behavior.

The HPA axis and stress hormones are also the focus of the work by Nakamura et al.. They employed salivary cortisol as an index of HPA axis functioning in the prenatal valproic acid-induced autism model (VPA) in marmosets, a species representing a robust preclinical model for the study of social stress and stress-induced social alterations. Their findings, aside from revealing similar diurnal changes in cortisol levels (i.e., lower in the afternoon than in the morning) in VPA-exposed and unexposed marmosets, also show that, similarly to human autism spectrum disorder (ASD), cortisol levels are increased throughout the day in VPA-exposed marmosets. This suggests that the VPA model in marmosets is a valid and useful tool for the study not only of the brain and behavior but also stress-related neuroendocrine alterations observed in human ASD.

Sex-dependent differences are the focus of 3 articles that use different rodent models of stress and/or psychiatric diseases. By using the prenatal exposure to VPA animal model for autism, Grgurevic tested the “male hypothesis” for this disorder, according to which autism is a manifestation of extreme male traits as evidenced by increased masculine behaviors, hypermasculinization of some brain regions, and alterations in androgen metabolism. A 2-fold approach is used, with behavioral testing assessing anxiety, sociability, and parental behavior in offspring of both sexes, and molecular analysis assessing brain expression of tyrosine hydroxylase (TH) in the anteroventral periventricular nucleus (AVPV) and plasma testosterone level. Findings revealed a significant sex-specific effect of VPA treatment, as a hypermasculinizing effect of VPA occurred specifically in male but not in female mice. Accordingly, VPA treatment decreased the number of AVPV TH positive cells in males only and elicited a lower quality of parental behavior in males than females.

Sex-dependent differences also emerged in the study by Scopano et al. that investigated the effects of the opioid peptide β-endorphin (β-E) in an animal model of early life adversity. They explored whether the effects of early stress on anxiety-like behaviors and alcohol reinforcement might be influenced by β-E. The authors tested adolescent and adult, female and male, wild-type C57BL/6J and β-E-deficient mice to investigate how maternal separation affects anxiety-like behavior in the open field and plus maze tests and initial reward to alcohol in a single-exposure conditioned place preference test. Findings showed that the impact of maternal separation changes over time, with more pronounced effects in adults than in adolescents, which was influenced by β-E in a sex-dependent manner.

In the last article by Bratzu et al. the effects of a socially enriched nesting condition (i.e., communal nesting, CN) and of an Early Social Isolation (ESI) protocol (milder than maternal separation) were assessed on motor, cognitive, and emotional domains during adolescence and adulthood in male and female rats reared in two different housing conditions. The hypothesis tested in this study was that an early-life socially enriched environment, i.e., the CN condition, may attenuate (if not fully prevent) the behavioral effects induced by pre-weaning social isolation. Results revealed that early-life social stress (ESI) induces a significant deficit in sensorimotor gating function in both adolescent males and females and in adult males, as indexed by PPI deficits. Additionally, ESI exposure produced compulsive burying behavior in adolescent male rats, with no changes on spontaneous motor behavior. Importantly, a protective effect of communal nesting was observed on both the sensorimotor gating deficit and compulsive burying behavior, with significant differences between males and females.

Collectively, these investigations underscore the significance of early adverse experiences as a substantial risk factor for the onset of psychiatric disorders in later life, emphasizing the multifaceted and sex-biased impact of stress during critical periods of brain development.

## Author contributions

LF: Conceptualization, Writing – original draft, Writing – review & editing. RF: Conceptualization, Writing – original draft, Writing – review & editing. FS: Conceptualization, Writing – original draft, Writing – review & editing.

